# Prevalence of medication-related problems and its predictors among cancer patients in Ethiopia: A systematic review and meta-analysis

**DOI:** 10.1371/journal.pone.0315818

**Published:** 2026-03-10

**Authors:** Malede Berihun Yismaw, Chernet Tafere, Bereket Bahiru Tefera, Adane Yehualaw, Desalegn Getnet Demsie, Kebede Feyisa, Zenaw Debasu Addisu, Zegaye Agmassie, Endalamaw Aschale, Tirsit Ketsela Zeleke, Rahel Belete Abebe, Eyob Ketema Bogale, Belayneh Kefale

**Affiliations:** 1 Department of Pharmacy, College of Medicine and Health Sciences, Bahr Dar University, Bahr Dar, Ethiopia; 2 Department of Pharmacy, College of Health Sciences, Debre Markos University, Debre Markos, Ethiopia; 3 Department of Clinical Pharmacy, School of Pharmacy, College of Medicine and Health Sciences, University of Gondar, Gondar, Ethiopia; 4 Department of Health Promotion and Behavioral Sciences, College of Medicine and Health Sciences, Bahr Dar University, Bahr Dar, Ethiopia; 5 School of Pharmacy and Pharmacology, University of Tasmania, Hobart, Tasmania, Australia; Medicine Information Services Unit, Pharmacy Services Division, ERITREA

## Abstract

**Background:**

Preventable medication-related problems intensify the risk associated with cancer care and no data was available to represent the burden of medication-related problems among cancer patients in Ethiopia. Hence, this study was aimed to estimate the pooled prevalence of medication-related problems and identify its predictors among cancer patients treated in Ethiopia.

**Methods:**

A systematic review and meta-analysis of studies retrieved from databases (Medline, EMBASE, Scopus, Cumulative Index to the Nursing and Allied Literature (CINAHL), Cochrane Library and Google Scholar) for relevant literature published before April, 2024 was made. We included observational studies conducted in Ethiopia that reported on the types, frequency, or risk factors of medication-related problems in cancer patients. Reviews, case reports, qualitative studies, and studies lacking relevant outcomes were excluded. The Newcastle-Ottawa quality assessment scale was used for quality assessment and the Egger’s regression test and the Galbraith plot were used to evaluate publication bias. The national prevalence of medication-related problems was estimated using a random-effects model meta-analysis. Moreover, subgroup analysis and meta-regression analyses were done to explore the reasons of statistical heterogeneity. The study protocol has been registered with PROSPERO under number CRD42024505218.

**Results:**

A total of 15 studies comprising of 3084 cancer patients were included in this study. The adjusted pooled prevalence of medication-related problems of cancer patients who experienced at least one medication-related problem was 48% [0.48 (95% CI: 0.39–0.57; I^2^ = 96%; p < 0.01)]. The study also revealed the pooled prevalence of non-adherence among the included studies to be 42% [0.42 (95% CI: 0.27–0.57; I^2^ = 97%; p < 0.01)]. The presence of comorbidities (AOR = 4.47, 95% CI: 3.26–5.69), complications (AOR = 5.78, 95% CI: 3.26–8.30) and polypharmacy (AOR = 3.75, 95% CI: 2.16–5.34) were found to be predictors of developing medication-related problems.

**Conclusion:**

This review found a high pooled prevalence of medication-related problems among cancer patients in Ethiopia, with predictors including comorbidities, complications and polypharmacy. About two-fifths of patients were not fully adherent to their prescribed cancer treatments. These findings highlight the need for targeted interventions to improve medication safety and adherence, and underscore the importance of future research to identify effective strategies for reducing MRPs in oncology settings.

## Introduction

Globally, cancer is the second leading cause of mortality after cardiovascular disease. According to the latest global report available as of 2024, GLOBOCAN estimates that cancer cases will rise from 19 million in 2020 to nearly 29 million by 2040. In 2020 there were nearly 10 million deaths from cancer globally, with this figure expected to increase to 16 million by 2040 [1].

Additionally, the trend of cancer incidence and deaths is forecasted to rise fastest in Africa compared to other regions. The cancer burden is projected to rise from 1.1 million cases and 711,429 deaths in 2020 to 2.1 million cases and 1.4 million cancer-related deaths in Africa by 2040 [2]. Furthermore, countries in sub-Saharan Africa, such as Ethiopia, are anticipated to see a markedly higher incidence and fatality rate. In Ethiopia, an estimated 80,334 numbers of cancer cases and 54,698 deaths occurred in 2022 [3].

Although substantial advancements including hormone therapy, hyperthermia, immunotherapy, photodynamic therapy, radiation therapy, stem cell therapy, surgery and targeted therapy are available for cancer management, chemotherapy remains the backbone of treatment in many cancer types [4]. People treated for cancer frequently experience a range of physical, psychological and social problems associated with chemotherapy that can reduce their quality of life (QoL). Cancer patients undergoing chemotherapy experience a range of medication-related problems (MRPs), and variations in prevalence exist between treatment centers [5]. Cancer patients are at increased risk of MRPs because they often receive multiple medications for cancer, comorbidities, and treatment-related complications. This polypharmacy, combined with the narrow therapeutic index of many anticancer agents, increases the likelihood of drug–drug and drug–disease interactions. Moreover, treatment-induced organ dysfunction, complex dosing regimens, and fragmented care across healthcare settings further heighten the risk of adverse drug events and suboptimal medication use [6].

MRPs have been defined as “an event or circumstance involving drug therapy that actually or potentially interferes with desired health outcomes” [7]. The major categories of MRPs encompass unnecessary drug therapy, need for additional drug therapy, ineffective drug therapy, too low dose, adverse drug reactions, too high dose, and medication non-adherence. A study of problems associated with medications conducted in Turkey showed that more than 80% of nurses made error during chemotherapy preparation or administration process. The result also depicted prescribing errors in about two-third of physicians and noncompliance to the prescribed patient by half of the patients [8].

Cancer therapy is complex, often exposing patients to a high risk of adverse drug reactions (ADRs), which are harmful or unintended responses to a drug at normal doses [9,10]. ADRs represent a major component of MRPs and can significantly compromise treatment effectiveness, patient safety, and quality of life [11]. A research done on ADR of chemotherapeutic agents showed prevalence in 58.6% of study subjects [12]. The research also emphasized the role of pharmacists on reporting and prospective benefits in preventing ADRs. ADRs are also a cause of readmission for 21% of cancer patients [13]. A study conducted in Belgium also reported ADRs in 41.1% of patients and was a cause for readmission of 10% of patients with in a month [14]. Pain is a common cancer-related adverse effect that significantly affects patients’ quality of life and requires careful management. Cancer pain increases the risk of MRPs due to the complexity of pain management [15]. For instance, an interventional study done on assessing MRPs among patients with cancer pain reported 78.6% of MRPs prevalence, with ineffective drug therapy (63.8%) being the most common followed by safety problem (36.2%).

Inadequate medication therapy imposes substantial economic burdens, with drug-related morbidity and mortality costing an estimated $528.4 billion in 2016, accounting for 16% of US healthcare expenditures. Expanding clinical pharmacists’ comprehensive medication management programs, including medication reconciliation, is critical for reducing MRPs and improving patient outcomes [16]. Evidence showed that pharmacist-led medication reconciliation decreased medication errors by 26% [17], and resolved up to 93.6% of all types of MRPs [18].

Different factors were reported as a risk factor for the occurrence of MRPs. According to a Belgian study, high level of comorbid clinical conditions, polypharmacy, the kind of hospital, and certain chemotherapies (platinum preparations) are associated with an increased incidence of MRPs in cancer patients [14]. While, other studies reported heavy work-load & staff shortage [8]; and being older, polypharmacy and presence of comorbidities [19] as the independent risk factors for MRPs.

MRPs are a major concern among cancer patients, as they can lead to ADRs, reduced treatment efficacy, increased healthcare costs, and poor patient outcomes. While individual studies on MRPs among cancer patients in Ethiopia exist [20–27], they have not been systematically synthesized to provide a comprehensive national overview of prevalence and predictors. Conducting a systematic review and meta-analysis provides a comprehensive synthesis of existing evidence, identifies the most common types of MRPs, and highlights factors that increase patients’ risk. Hence, the present systematic review and meta-analysis aimed to synthesize, summarize and critique original studies done to assess MRPs among cancer patients in Ethiopia and address the following research questions:

- What is the pooled prevalence of MRPs among cancer patients treated in Ethiopia?
- What are the common types of MRPs that cancer patients encounter?
- What are the risk factors contributed for the existence of MRPs?

The findings of this study is helpful to inform healthcare professionals, policymakers, and researchers about the scope of MRPs in Ethiopian cancer care, guiding the development of targeted interventions to optimize medication use, enhance patient safety, and improve overall treatment outcomes.

## Methods

### Review protocol

The protocol was registered in the International Prospective Register of Systematic Review (PROSPERO) under registration number CRD42024505218. This systematic review was carried out and reported using the Preferred Reporting Items for Systematic review and Meta-analysis (PRISMA) checklist [28]. The filled form is shown in S1 Checklist. A PRISMA flow diagram was employed to illustrate the steps involved in identification, eligibility screening, and final inclusion.

### Data source and search strategy

Studies that assess MRPs among cancer patients in Ethiopia were obtained from databases including Medline, EMBASE, Scopus, Cumulative Index to the Nursing and Allied Literature (CINAHL) and Cochrane Library. In addition, grey literature and papers that were not indexed in major databases were sought via Google Scholar. A systematic literature search was made using the following search terms: (“drug-related side effects and adverse reactions” OR “adverse drug event*” OR “adverse drug reactions” OR “drug interactions” OR “Drug Interactions” OR “medication adherence” OR “medication non-adherence” OR “medication noncompliance” OR “Drug adherence” OR “Drug non-adherence” OR “Drug noncompliance” OR “medication errors” OR “drug errors OR “Drug related problem*” OR “Drug therapy problem*” OR “Medication-related problem*” OR “Medication therapy problem*” OR “Medication related harm*” OR “Drug related harm*” OR “Chemotherapy problem*” OR “chemotherapy-induced nausea and vomiting” OR “Inappropriate medication use” OR “Inappropriate drug use” AND (“neoplasms” OR “tumors” OR “cancer” OR “malignancy” AND (“Ethiopia”). This systematic review and meta-analysis included all relevant published studies conducted before April, 2024.

### Eligibility criteria

All retrieved studies’ titles, abstracts, and/or full-text were evaluated for eligibility. Studies that evaluated the types, frequencies, and/or risk factors linked to MRPs as primary or secondary outcomes in patients receiving cancer treatment in Ethiopian outpatient or inpatient settings were included.

### Inclusion criteria

- Observational studies (cross-sectional, case-control, and cohort studies) with original data reporting MRPs among cancer patients in Ethiopia, regardless of cancer type, stage, treatment modality, or age.
- Unpublished studies and grey literature, including theses and dissertations, were considered.
- Studies reported the types, frequency, or risk factors of MRPs as outcome variables.
- Literature written in English or with an additional English translation was included
- Any published literature with a publication date before April, 2024.

### Exclusion criteria

- Articles with personal comments, conference papers, editorial reports, letters to the editor, randomized controlled trials (RCTs), systematic reviews, records with missing outcomes of interest, studies without accessible full texts and inadequate information were excluded.
- Case studies, case series, and qualitative studies were also excluded.

### Study selection and data extraction

All studies obtained using search strategies were exported to EndNote citation manager, and the duplicates were removed. Finally, all studies were exported to a Microsoft Excel spreadsheet. The titles and abstracts of studies retrieved and those from unpublished sources were screened to identify studies that satisfy the inclusion criteria. Then, the full text of potentially eligible studies was assessed. The authors’ name, publication year, study design, study region, study setting, target population, focus of the study, sample size, attributes of MPRs, and associated factors were included in the data extraction format. Data were independently extracted by two reviewers (M.B.Y and B.K.) using a pre-designed extraction form that was piloted before use. Any missing or unclear data were addressed by contacting the original study investigators to ensure completeness and accuracy.

### Quality assessment and risk of bias

The Newcastle-Ottawa quality assessment scale (NOS) adapted for observational studies was used to assess the quality of each original study [29]. This scale was used to evaluate the internal and external validity, risk of bias, and methodological quality of each original study included. The quality assessment tool is divided into three sections. The first section concentrated on each original study’s methodological quality, such as objectives, sample size, and sampling technique. This section was graded on a 5-star scale. The tool’s second section evaluates study comparability and assigns a star rating out of two. The third section of the tool evaluates the outcome measures and data analysis and assigns a star rating out of three. The NOS leveled a study as good if the score is 7–8, and very good for scores 9–10.

The review and meta-analysis included studies with a score of 5 or higher. The articles’ quality was assessed by three authors (MBY, CT and DGD). The authors then compared the scores assigned to each study. Discrepancies in ratings were resolved through consensus.

### Outcome variables

The primary outcome of this systematic review and meta-analysis was estimating the pooled prevalence of MRPs among patients receiving cancer treatment in Ethiopia. Prevalence of MRPs was directly taken from the reports of the primary studies or calculated by dividing the number of patients experienced MRPs to the total number of study participants. While, variables reported as risk factor for the existence of MRPs and pooled prevalence of non-adherence were incorporated as a secondary outcome of interest.

### Statistical analysis

The extracted data were imported from the Microsoft Excel data extraction format to R software Version 4.3.2 for further analysis. The pooled national prevalence of MRPs was estimated using a random-effects meta-analysis model [30]. The heterogeneity of primary studies was checked using I^2^ test. Based on the test result, a random-effects meta-analysis model was used to estimate Der Simonian and Laird’s pooled prevalence of outcomes. In addition, subgroup analyses were performed across the study designs and study areas in order to identify the cause of heterogeneity apart from random variation across the included studies. Potential publication bias had also been examined through visual assessment of the funnel plot and Egger’s test. A statistical test with a p value of less than 0.05 was considered significant in all cases.

## Results

**Fig 1:**
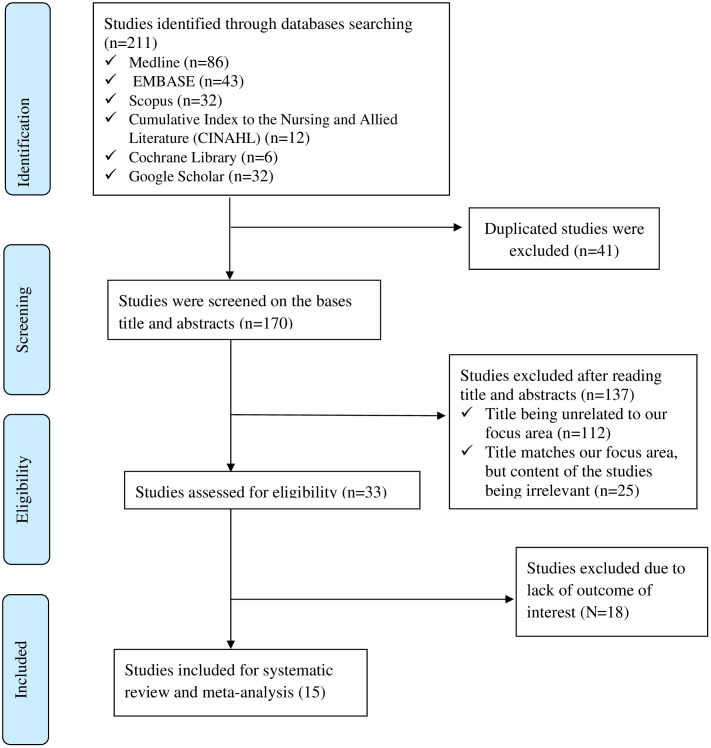
PRISMA flow diagram describing the selection of studies for systematic review and meta-analysis of medication-related problems among cancer patients in Ethiopia.

A total of 211 articles were retrieved using the search terms in varied databases (**Fig 1**). Of these, 41 studies were excluded as duplicates, 137 were excluded for not meeting the inclusion criteria—including those with the wrong population (non-cancer patients or studies outside Ethiopia) or an inappropriate study design (e.g., qualitative studies and reviews)—and 18 were excluded for lacking outcomes of interest. Finally, only 15 studies were eligible for inclusion in the current meta-analysis. The full lists of articles screened and included were shown in S1 Table.

### Characteristics of the included studies

All original studies assessing MRPs in any cancer type of all age groups were included for further analysis. The majority (9, 60%) of the included studies [19,22–24,31–35] employed cohort research design, while the rest [20,21,25–27,36] were cross-sectional type. Five studies (33.33%) [21,23,24,26,36] assessed MRPs in any type of cancer, and only one study [32] was conducted on chronic myeloid leukemia. Nearly half of the studies (53.33) [19,21,26,27,33–35] were conducted in Amhara region, Ethiopia. Only two of the studies [23,26] were conducted among childhood cancer patients. The sample size had a significant variation ranging from 26 in a study conducted at Aira Hospital of Oromia reginal state [22] to 433 in a study conducted at Felege Hiwot and Dessie comprehensive specialized hospitals [21]. The mean MRPs per patient ranged from 0.16 [25] to 1.9 [27] (**Table 1**). The data set used for the review is available in S2 Table.

**Table 1:** General characteristics of studies conducted on medication-related problems among cancer patients in Ethiopia.

Author	Publication year	Study design	Medical condition	Study area	Study setting	Population group	Aim of study	Sample size	Prevalence/%	Number of patients with MRPs	Total number of MRPs	Mean MRPs
Bekalu et al. [21]	2023	CSP	All Ca*	Amhara	OPD	adult	non-adherence	433	57.7	250	250	0.58
Reibold et al. [22]	2021	CoP	Breast ca*	Oromia	OPD	adult	non-adherence	26	65.4	17	17	0.65
Yismaw et al. [23]	2020	CoP	All ca	TASH, AA	In-patient	pediatrics	MRPs	156	68.6	107	257	1.65
Hassen et al. [25]	2022	CSP	Breast ca*	TASH, AA	OPD	adult	non-adherence	164	16.5	27	27	0.16
Yitayih et al. [20]	2015	CSP	cervical cancer	TASH, AA	OPD	adult	non-adherence	314	30.3	95	95	0.3
Workalemahu et al. [26]	2020	CSR	All Ca*	Amhara	In-patient	pediatric	ADR	287	41.5	119	119	0.42
Dessalegn et al. [31]	2023	CoP	SM	AA	In-patient	adult	ADR	98	70.7	65	65	0.71
Degu et al. [27]	2021	CSP	Breast ca*	Amhara	in-patient&OPD	adult	MRPs	107	71.03	76	203	1.9
Fentie et al. [32]	2019	CoP	CML	AA	OPD	adult	non-adherence	147	55.1	81	81	0.55
Kefale et al. [33]	2023a	CoR	Colorectal ca*	Amhara	in-patient&OPD	adult	MRPs	143	53.1	76	186	1.3
Kefale et al. [34]	2023b	CoR	cervical ca*	Amhara	in-patient&OPD	adult	MRPs	124	59.7	74	168	1.35
kefale et al. [19]	2022a	CoR	colorectal ca*	Amhara	in-patient&OPD	adult	MRPs	150	48.7	73	153	1.02
kefale et al. [35]	2022b	CoR	cervical ca*	Amhara	in-patient&OPD	adult	MRPs	184	50.5	93	216	1.17
Belachew et al. [36]	2016	CSP	All Ca*	Amhara	in-patient&OPD	adult	ADR	384	52.9	203	815	2.12
Sisay et al. [24]	2015	CoR	All Ca*	AA	in-patient	adult	MRPs	367	74.7	274	474	1.29

AA; Addis Ababa; ADR: adverse drug reaction; ca*: cancer; CML: chronic myeloid leukemia; CoP: prospective cohort; CoR: retrospective cohort; CSP: prospective cross-sectional; CSR: retrospective cross-sectional; MRP: medication-related problem; OPD: outpatient department; and SM: solid malignancies; TASH: Tikur Anbessa Specialized Hospital.

### Quality assessment of included studies

The current meta-analysis includes studies done using cross-sectional and cohort research designs. Based on the NOS, the qualities of the studies was rated as good for four studies and very good for eleven studies. Details on the scores of individual studies are illustrated below (**Table 2**).

**Table 2:** Quality assessments of studies conducted on medication-related problems among cancer patients in Ethiopia based on the Newcastle-Ottawa Scale.

Study	Selection	Comparability	Outcome	Total
Bekalu et al. 2023	5	2	3	10
Reibold et al. 2021	5	1	2	8
Yismaw et al. 2020	5	1	3	9
Hassen et al. 2022	4	2	2	8
Yitayih et al. 2015	4	1	2	7
Workalemahu et al. 2022	5	1	3	9
Dessalegn et al. 2023	5	2	3	10
Degu et al. 2021	5	2	2	9
Fentie et al. 2019	5	2	3	10
Kefale et al. 2023a	5	2	3	10
Kefale et al. 2023b	5	2	3	10
kefale et al. 2022a	5	2	3	10
kefale et al. 2022b	5	2	3	10
Belachew et al. 2016	4	2	2	8
Sisay et al. 2015	5	2	2	9

### Prevalence of MRPs

**Fig 2:**
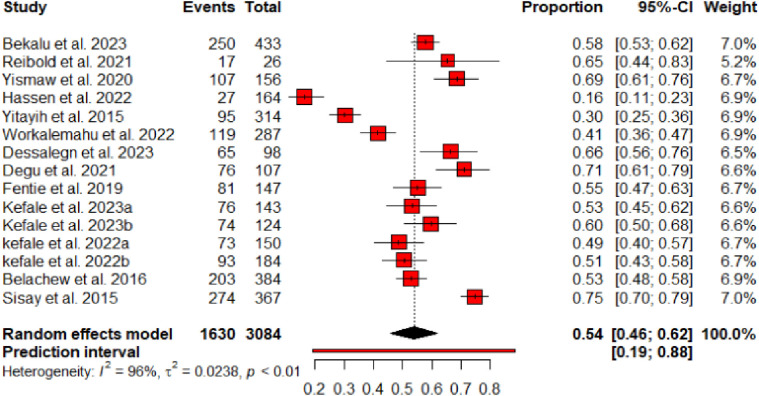
Pooled prevalence of MRPs among cancer patients in Ethiopia.

The prevalence of MRPs ranges from 16.5% in a study assessed non-adherence of breast cancer treatments at TASH [25] to 74.7% in a study assessed MRPs among cancer patients taking chemotherapy at the same hospital [24]. Overall, the current meta-analysis utilized data of 15 studies with 3084 cancer patients. Of the included patients, 1630 of them encountered at least one MRP and gives a pooled prevalence of 54% [0.54 (95% CI 0.46–0.62; I^2^ = 96%; p < 0.01)] (**Fig 2**).

### Subgroup analysis

**Fig 3:**
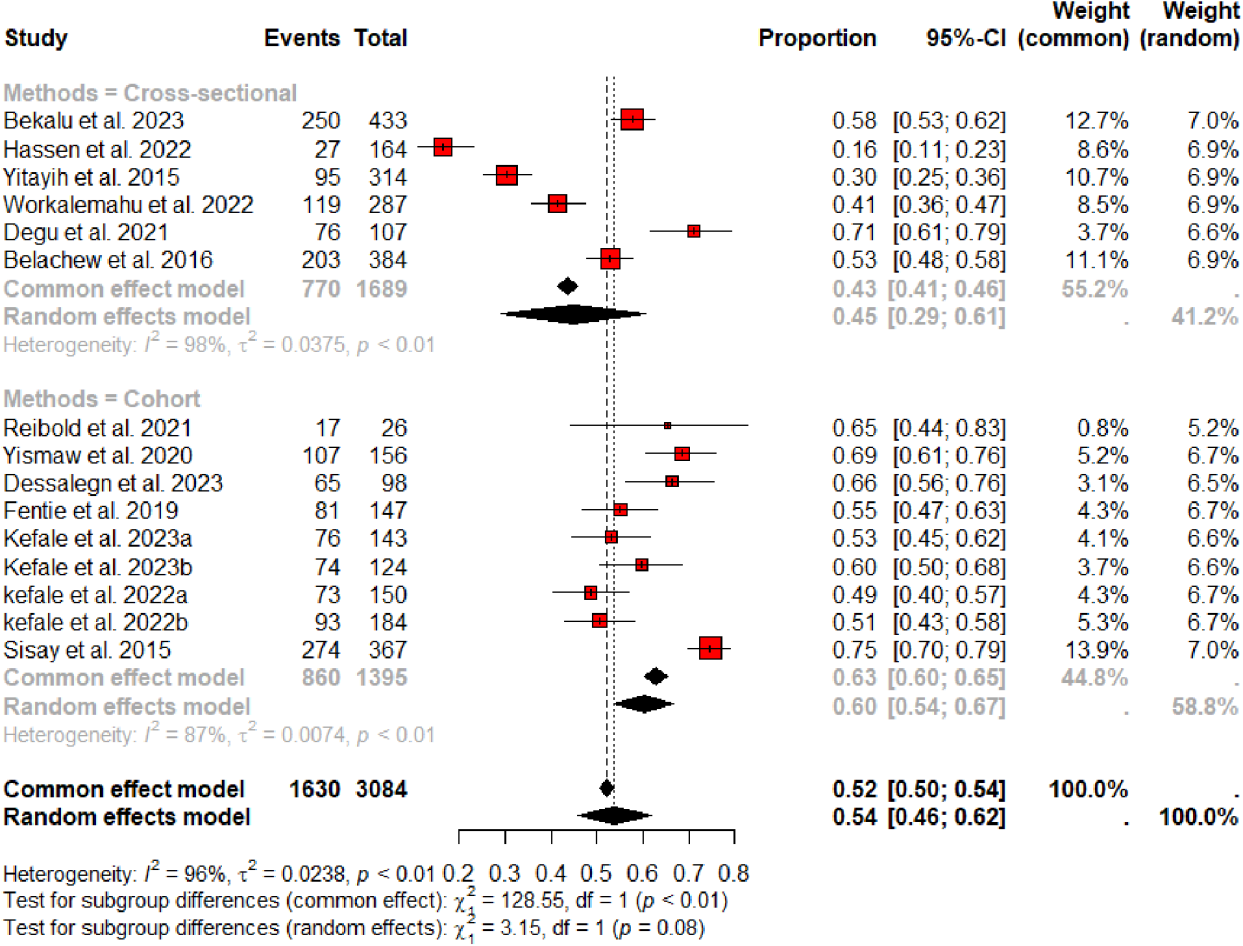
Sub-group analysis of MRPs across study designs.

We conducted subgroup analysis to explore sources of heterogeneity and to evaluate whether there was significant difference in magnitude of MRPs across the study designs used (cross-sectional and cohort). The result showed that the pooled prevalence of MRPs among cross-sectional studies [20,21,25–27,36] was 45% [0.45 (95% CI: 0.29–0.61; I^2^ = 98%; p < 0.01)], while it was 60% [0.60 (95% CI: 0.54–0.67; I^2^ = 87%; p < 0.01)] among studies [19,22–24,31–35] employed cohort research designs and there was no significant difference across study designs (**Fig 3**).

### Characterization of MRPs

The included studies reported either one type of MRP or more. Ten studies [19,23,24,26,27,31,33–36] included the magnitude of ADRs. Among studies reported ADRs, a study conducted by Dessalegn et al., 2023 [31] showed a higher prevalence of ADRs among the study participants. Seven of the studies [19,23,24,27,33–35] reported the proportion of unnecessary medication uses and patients needing additional drug therapy. While only two studies [23,27] identified the prevalence of ineffective drug therapy (**Table 3**).

**Table 3:** Types of MRPs reported among studies included in the meta-analysis.

Study	Unnecessary drug therapy (%)	Needs additional therapy (%)	Ineffective drug (%)	Dosage too low (%)	ADR (%)	Dosage too high (%)	Non-adherence (%)	DDI (%)
Bekalu et al. 2023	–	–	–	–	–	–	57.7	–
Reibold et al. 2021	–	–	–	–	–	–	65.4	–
Yismaw et al. 2020*	9.7	27.2	4.3	23.3	5.5	16	14	–
Hassen et al. 2022	–	–	–	–	–	–	16.5	–
Yitayih et al. 2015	–	–	–	–	–	–	30.3	–
Workalemahu et al. 2022	–	–	–	–	41.5	–	–	–
Dessalegn et al. 2023	–	–	–	–	70.7	–	–	–
Degu et al. 2021	7.5	45.8	18.7	7.5	48.6	12.1	32.5	16.8
Fentie et al. 2019	–	–	–	–	–	–	55.1	–
Kefale et al. 2023a*	7	29.6	–	22	18.3	4.8	–	18.3
Kefale et al. 2023b*	10.7	22.6	–	24.4	22	4.2	–	16.1
kefale et al. 2022a*	3.1	17	–	11.1	32	3.3	–	32.7
kefale et al. 2022b*	7.4	22.2	–	15.7	27.3	2.3	–	25
Belachew et al. 2016	–	–	–	–	52.9		–	–
Sisay et al. 2015	16.9	8.2	–	37.9a	45.5	37.9a	–	3

ADR: Adverse drug reaction; DDI: Drug-drug interaction;* proportion from total MRPs.

**Fig 4:**
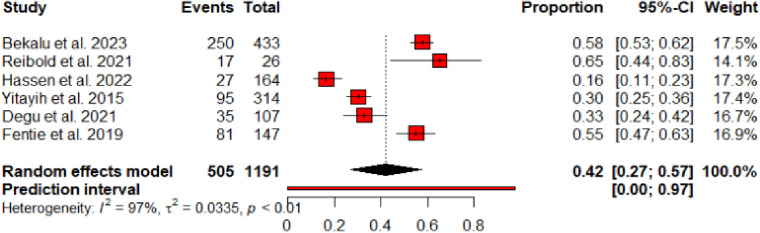
Pooled prevalence of non-adherence among studies reported the proportion of non-adherent individuals from the sample population.

Of the fifteen studies included, seven of them [20–23,25,27,32] reported the magnitude of non-adherence to chemotherapeutic agents among adult cancer patients. Among the seven studies that reported non-adherence, the study by Yismaw et al. [23] did not present the prevalence of non-adherence among participants; rather, it reported the proportion of non-adherence as part of the total drug-related problems encountered. Consequently, the rest six studies [20–22,25,27,32] were used to analyze the pooled prevalence of non-adherence. Accordingly, the pooled prevalence of non-adherence among the included six studies was found to be 42% [0.42 (95% CI: 0.27–0.57; I^2^ = 97%; p < 0.01)] (**Fig 4**).

### Predictors of MRPs

With related to risk factors for the existence of MRPs, eleven of them [19,21,24–27,32–36] analyzed inferential statistics and reported significant predictors for the occurrence of MRPS. Majority of the studies [19,21,24,27,32,33,35] reported that comorbidity was significantly associated with outcome of interest. Seven studies [19,24,26,33–36] also showed the association between polypharmacy and MRPs. Cancer stage [33,34], age [19,34,36] and the development of complications [21,32,35] were also identified as risk factors (**Table 4**).

**Table 4:** Individual studies report of factors associated with the occurrence of MRPs among cancer patients in Ethiopia.

Studies	Aim of study	Risk factors
Bekalu et al. 2023	non-adherence	Family history of cancer, being female, comorbidity, complications
Hassen et al. 2022	non-adherence	Distance from referral center and course/regimen of chemotherapy
Workalemahu et al. 2020	ADR	Polypharmacy and course/regimen of chemotherapy
Degu et al. 2021	MRPs	Comorbidities, course/regimen of chemotherapy
Fentie et al. 2019	non-adherence	Occupation, income, residence, complications and comorbidities
Kefale et al. 2023a	MRPs	Cancer stage, comorbidities, complications and polypharmacy
Kefale et al. 2023b	MRPs	Age, cancer stage, and polypharmacy
kefale et al. 2022a	MRPs	Age, comorbidities and polypharmacy
kefale et al. 2022b	MRPs	Complications, comorbidities and polypharmacy
Belachew et al. 2016	ADR	Age, polypharmacy and dose of chemotherapy
Sisay et al. 2015	MRPs	Hospital stay, comorbidities and polypharmacy

The study also identified the pooled effect of predictors for the occurrence of MRPs. Only studies reported both adjusted odds ratio (AOR) with 95% confidence interval (CI) were included to identify pooled effect of predictors for MRPs. Thus, among eleven studies (13, 15, 18–21, 26–30) reported predictors for the occurrence of MRPS, only eight of them (13, 15, 18, 20, 26–29) were used to estimate the pooled effects of predictors. Studies done by Hassen et al.2022 (19) and Belachew et al.2016 (30) were excluded due to unavailability of AOR with 95% CI. A study done by Degu et al.2021 (21) also did not report 95% CI of the AOR and excluded in the pooled predictors analysis. Comorbidities, complications, polypharmacy, advanced cancer stage, and age were candidate variables. Accordingly, comorbidities (AOR = 4.47, 95% CI = 3.26–5.69, p < 0.001), complications (AOR = 5.78, 95% CI = 3.26–8.30, p < 0.001) and polypharmacy (AOR = 3.75, 95% CI = 2.16–5.34, p < 0.001) were found to increase the odds of developing MRPs significantly (**Table 5**).

**Table 5:** Pooled effect of predictors for the development of MRPs among cancer patients in Ethiopia.

Predictors	Number of studies	AOR	95% CI	P-value
Comorbidities	Six [19,21,24,32,33,35]	4.47	3.26-5.69	P < 0.001*
Complications	Four [21,32,33,35]	5.78	3.26-8.30	P < 0.001*
Polypharmacy	Six [19,24,26,33–35]	3.75	2.16-5.34	P < 0.001*
Advanced cancer stage	Two [33,34]	18.05	(−0.85)-36.96	P = 0.06
Being elderly	Two [19,34]	9.60	(−1.65)-20.85	P = 0.09

* Statistically significant.

### Publication bias

**Fig 5:**
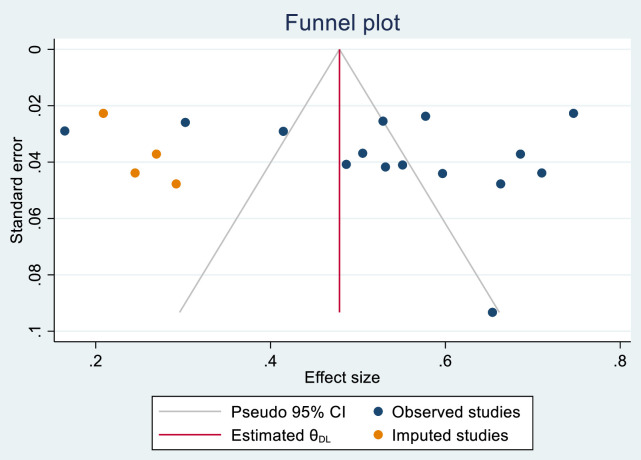
Funnel plot showing event by the standard error of event for publication bias.

The funnel plot analysis (prevalence vs. standard error of prevalence) was done to assess publication bias of included studies (**Fig 5**). Furthermore, Begg’s correlation and Egger’s regression test were used to screen for publication bias. The results of both tests (p = 0.488 and p = 0.234, respectively) did not support the existence of publication bias among the included studies. But, when we run the non-parametric trim and fill analysis of publication bias, we got 4 imputed studies. Accordingly, the adjusted pooled prevalence was found to be 48% [0.48 (95% CI: 0.39–0.57)].

## Discussion

This systematic review and meta-analysis provides the first pooled evidence on the prevalence and predictors of MRPs among cancer patients in Ethiopia. Our findings indicated that MRPs are a substantial and persistent challenge in oncology care, reflecting the complexity of cancer pharmacotherapy, high pill burden, and the frequent need for individualized treatment adjustments. The observed prevalence underscores the critical implications of MRPs for patient safety, treatment effectiveness, and healthcare resource utilization. Interpreting these results in the context of existing literature allowed us to explore underlying factors, compare trends with other settings, and identify priority areas for intervention to optimize pharmacotherapy in Ethiopian cancer care.

A huge variation in the prevalence of MRPs among studies was reported. For instance, a study done by Hassen et al [25] reported a relatively low (16.5%) prevalence. On the other hand, Sisay et al [24] reported a high prevalence (74.7%), but analyzed all types of MRPs among all types of adult cancer diagnoses. The present systematic review and meta-analysis revealed that the adjusted pooled prevalence of MRPs among cancer patients in Ethiopia to be 48% [0.48 (95% CI: 0.39–0.57)]. Similarly, a study done in Northern Cyprus showed a 53% prevalence of MRPs among cancer patients [37]. On the contrary, other studies conducted among cancer patients in Canada [38] and India [39] showed a MRP prevalence of 62.1% and 74%, respectively. The low prevalence in our study might be due to the fact that our study also included adult patients and cross-sectional research designs unlike the two studies [38,39] which were done among geriatric patients and employed a prospective follow-up research design that leads to an increase in the occurrence of MRPs as compared to the current study. Elderly patients are at increased risk of MRPs due to plenty of reasons such as decline in organ functions, comorbid medical conditions and polypharmacy [40,41].

Surprisingly, a study conducted among cervical cancer patients at Kenyatta National Hospital, Kenya showed a significantly higher (93.8%) prevalence of MRPs [42]. The significant variation observed in our study may be attributed to the non-reporting of mild, clinically insignificant drug–drug interactions in the included studies, which would otherwise increase the reported prevalence of MRPs. The heterogeneity might also arise from variations in the study population, MRPs classification, and the composition of healthcare providers in the study settings. Researchers should focus on clinically significant MRPs which majorly determines the treatment outcome, quality of life and healthcare expenditure.

Although the subgroup analysis showed no significant difference across study designs, the pooled prevalence of MRPs among studies [19,22–24,31–35] employed cohort research designs (60%) was higher than studies utilized cross-sectional research design [20,21,25–27,36]. The higher occurrence of MRPs in cohort studies could be explained by their longer follow-up periods, during which more MRPs are likely to develop, unlike cross-sectional studies that capture data at only one moment in time. A subgroup analysis did not show a significant difference in magnitude of MRPs across the study areas. Hence, we conclude that the observed heterogeneity might be caused by random variation as the current meta-analysis included the limited number of publications available.

Recognizing the many types of MRPs adds value to efforts to both avoid and address their impacts. The included studies reported either one type of MRP or more. Two-third of the included studies [19,23,24,26,27,31,33–36] reported the magnitude of ADRs. Among studies reported ADRs, a study conducted by Dessalegn et al [31] showed a higher (70.7%) prevalence of ADRs among the study participants. Dessalegn et al. [31] followed patients until completion of five chemotherapy cycles, which likely increased the probability of detecting more ADRs and thus contributed to the higher prevalence observed. Likewise, a prospective observational survey conducted in India reported an ADR prevalence of 58.6%, with half of the ADRs deemed to be preventable [12]. ADRs are common among patients receiving chemotherapeutic agents, ranging from mild reactions like nausea and vomiting to severe effects such as bone marrow suppression [43–45]. Therefore, developing preventative measures is essential for the early detection and management of ADRs.

Six studies [20–22,25,27,32] reported the prevalence of non-adherence among study subjects resulting in a pooled prevalence of non-adherence to be 42% [0.42 (95% CI 0.27–0.57; p < 0.01)]. Consistent to our study, a non-adherence of 32.1% was reported in a study [42] conducted at Kenyatta National Hospital, Kenya. On the contrary, a significantly higher (80.9%) of non-adherence prevalence was reported among breast cancer patients in Nigeria [46]. The higher prevalence observed in Nigeria may be partly due to the 10-year retrospective health record data, as longer follow-up periods tend to capture a cumulative increase in reported non-adherence.

In Ethiopia, cost and availability are factors contributed to non-adherence. For instance, a study done on price, availability and affordability of cytotoxic drugs at in Addis Ababa, Ethiopia showed that drugs for cancer treatment are unavailable and the few available medicines are also unaffordable [47]. Likewise, a study from Nigeria [48] identified that low economic status, adverse effects of cytotoxic medications (such as weight loss and hair loss), length of treatment, and an unfavorable clinic visit hours as contributors for non-adherence among cancer patients. Non-adherence to cancer treatments may lead to adverse clinical outcomes as well as higher rates of morbidity and mortality [49–53]. Non-adherent patients often incur significant financial, medical, and psychological costs. Adherence to the prescribed medications and recommended non pharmacological management modalities is a key for achieving the goal of treatment [54–56]. It has been suggested that patients and healthcare providers should develop a favorable relationship in order to support successful and long-term medication adherence.

Identifying patients at high risk of MRPs can help to target risky groups and prevent associated risks. Accordingly, we investigated independent factors from individual studies and analyzed pooled effects of predictors. The pooled predictor analysis showed that patients with preexisting comorbidities are 4.47 times at high risk of developing MRPs. Furthermore, patients who developed complications and those taking multiple medications had a 5.78-fold and 3.75-fold higher risk of experiencing MRPs, respectively. In line with the present study, a retrospective study done in Brazil showed that comorbidities and polypharmacy are associated with increased risk of MRPs among cancer patients [57]. Studies done among cancer patients in Canada [38] and Kenya [42] also identified that polypharmacy was an independent risk factors for the development of MRPs. Patients with preexisting comorbidities and complications need multiple medications. The use of these multiple drugs leads to drug-drug interactions, non-adherence, safety and efficacy problems. As a result, the risk of developing MRPs will be increased in these groups of populations.

Studies conducted elsewhere showed that clinical pharmacists’ intervention including Meducation Therapy Management Services (MTM) has been shown a reduction in MRPs and an increase in the quality of life of cancer patients [37,57–59]. Accordingly, integration of clinical pharmacists in cancer care can decrease healthcare cost, drug related morbidity and mortality as well as improve quality of life of patients living with cancer.

### Strength and limitation

The included studies varied in terms of their design, population group, prescribed medications, type of cancer diagnosis, and the health care settings in which they were conducted. As a result, we were unable to draw conclusions about some data, such as the types of medications that most frequently contributed to the occurrence of MRPs and the priority areas for MRP prevention. Additionally, most included studies reported multiple types of MRPs without providing separate prevalence estimates for each category, which made it infeasible to perform a meaningful subgroup analysis in this aspect. Nevertheless, despite these limitations, the review findings offered a novel perspective on the scope of the issue at the national level.

## Conclusion

MRPs were highly prevalent among cancer patients in Ethiopia, with adverse drug reactions emerging as the most frequently reported concern, underscoring the need for careful monitoring and management of therapy in this population. About two-fifths of patients were not fully adherent to their prescribed cancer treatments. The existence of comorbid medical conditions, complications and polypharmacy were attributed for the occurrence of MRPs. Given the high prevalence of MRPs and identified risk factors, integrating clinical pharmacists within cancer care is critical to improve treatment outcomes. Addressing barriers to adherence, particularly by ensuring drug availability and affordability, is essential for promoting sustained medication use among cancer patients. Furthermore, future research should focus on the impact of clinically significant MRPs on treatment outcomes and quality of life. Expanding studies to include multicenter designs and a broader range of patient demographics will provide more comprehensive data to guide targeted MRP prevention and management strategies.

## Supporting information

S1 Checklist Completed PRISMA 2020 checklist. (DOCX)

S1 Table Full lists of articles screened and included in the review. (DOCX)

S2 Table Data set showing the data extracted from the included studies. (DOCX)
