# Supplementary material for: Prevalence of medication-related problems and its predictors among cancer patients in Ethiopia: A systematic review and meta-analysis
Source: PLoS One. 2026 Mar 10;21(3):e0315818. doi: 10.1371/journal.pone.0315818 (PMC12974847; doi:10.1371/journal.pone.0315818)
Supplement: S1 Table — (DOCX) [file pone.0315818.s002.docx]

| Author | year | design | medical condition | study area | study setting | Population group | aim of study | sample size | prevalence/% | number of patients with DRPs | Total number of DRPs | mean DRPs | %Unnecessary drug therapy | %Needs additional therapy | %Ineffective drug | %Dosage too low | %ADR | %Dosage too high | %nonadherence | DDI |
| --- | --- | --- | --- | --- | --- | --- | --- | --- | --- | --- | --- | --- | --- | --- | --- | --- | --- | --- | --- | --- |
| Bekalu et al | 2023 | cross-sectional-pros | All Ca | Amhara | OPD | adult | non-adherence | 433 | 57.7 | 250 | 250 | 0.58 |  |  |  |  |  |  | 57.7 |  |
| Reibold et al | 2021 | cohort-pros | Breast ca | Oromia | OPD | adult | non-adherence | 26 | 65.4 | 17 | 17 | 0.65 |  |  |  |  |  |  | 65.4 |  |
| Yismaw et al | 2020 | cohort-pros | All ca | TASH, AA | In-patient | pediatrics | MRPs | 156 | 68.6 | 107 | 257 | 1.65 | 9.7 | 27.2 | 4.3 | 23.3 | 5.5 | 16 | 14 |  |
| Hassen et al | 2022 | cross-sectional-pros | Breast ca | TASH, AA | OPD | adult | non-adherence | 164 | 16.5 | 27 | 27 | 0.16 |  |  |  |  |  |  | 16.5 |  |
| Yitayih et al | 2015 | cross-sectional-pros | cervical cancer | TASH, AA | OPD | adult | non-adherence | 314 | 30.3 | 95 | 95 | 0.3 |  |  |  |  |  |  | 30.3 |  |
| Workalemahu et al | 2020 | cross-sectional-retro | All Ca | Amhara | In-patient | pediatrics | ADR | 287 | 41.5 | 119 | 119 | 0.42 |  |  |  |  | 41.5 |  |  |  |
| Dessalegn et al | 2023 | cohort-pros | Solid malignancy | SPHMMC,AA | In-patient | adult | ADR | 98 | 70.7 | 65 | 65 | 0.71 |  |  |  |  | 70.7 |  |  |  |
| Degu et al | 2021 | cross-sectional-pros | Breast ca | Amhara | in-patient&OPD | adult | MRPs | 107 | 71.03 | 76 | 203 | 1.9 | 7.5 | 45.8 | 18.7 | 7.5 | 48.6 | 12.1 | 32.5 | 16.8 |
| Fentie et al | 2019 | cohort-pros | CML | TASH, AA | OPD | adult | non-adherence | 147 | 55.1 | 81 | 81 | 0.55 |  |  |  |  |  |  | 55.1 |  |
| Kefale et al | 2023a | cohort-retro | Colorectal ca | Amhara | in-patient&OPD | adult | MRPs | 143 | 53.1 | 76 | 186 | 1.3 | 7 | 29.6 |  | 22 | 18.3 | 4.8 |  | 18.3 |
| Kefale et al | 2023b | cohort-retro | cervical ca | Amhara | in-patient&OPD | adult | MRPs | 124 | 59.7 | 74 | 168 | 1.35 | 10.7 | 22.6 |  | 24.4 | 22 | 4.2 |  | 16.1 |
| kefale et al | 2022a | cohort-retro | colorectal ca | Amhara | in-patient&OPD | adult | MRPs | 150 | 48.7 | 73 | 153 | 1.02 | 3.1 | 17 |  | 11.1 | 32 | 3.3 |  | 32.7 |
| kefale et al | 2022b | cohort-retro | cervical ca | Amhara | in-patient&OPD | adult | MRPs | 184 | 50.5 | 93 | 216 | 1.17 | 7.4 | 22.2 |  | 15.7 | 27.3 | 2.3 |  | 25 |
| Belachew et al | 2016 | cross-sectional-pros | All Ca | Amhara | in-patient&OPD | adult | ADR | 384 | 52.9 | 203 | 815 | 2.12 |  |  |  |  | 52.9 |  |  |  |
| Sisay et al | 2015 | cohort-retro | All Ca | tASH, AA | in-patient | adult | MRPs | 367 | 74.7 | 274 | 474 | 1.29 | 16.9 | 8.2 |  | 37.9a | 45.5 | 37.9a |  | 3 |
